# Immediate Extubation Following Living Donor Liver Transplantation: Feasibility, Safety, and Impact on Early Outcomes

**DOI:** 10.1155/ijh/3504959

**Published:** 2026-09-29

**Authors:** Muhammad Nauman-ul-Haq, Muhammad Zakariya Amjad, Saad Ahmed Naveed, Haseeb Haider Zia, Nusrat Yar Khan, Maliha Aziz, Abid Ilyas, Ayesha Junaid, Nasir Ayub Khan, Abu Bakar Hafeez Bhatti

**Affiliations:** ^1^ Department of HPB Surgery and Liver Transplantation, Shifa International Hospital, Islamabad, Pakistan, shifa.com.pk; ^2^ Department of Anesthesiology, Shifa International Hospital, Islamabad, Pakistan, shifa.com.pk; ^3^ Shifa Tameer-e-Millat University, Islamabad, Pakistan, stmu.edu.pk; ^4^ Clinical Research Center, Shifa International Hospital, Islamabad, Pakistan, shifa.com.pk; ^5^ Department of Surgical Critical Care, Shifa International Hospital, Islamabad, Pakistan, shifa.com.pk; ^6^ Department of Hematology, Shifa International Hospital, Islamabad, Pakistan, shifa.com.pk

**Keywords:** extubation, intensive care unit, living donor liver transplantation, model for end-stage liver disease, survival

## Abstract

**Background:**

Early extubation is a key component of enhanced recovery pathways but remains underutilized in living donor liver transplantation. This single‐center retrospective cohort study evaluates the feasibility, safety, and clinical outcomes of immediate operating room extubation using standardized intraoperative physiological criteria.

**Methods:**

A total of 158 consecutive adult LDLT recipients meeting standardized intraoperative extubation criteria were included and categorized into immediate (OR) and delayed (ICU) extubation groups. Primary outcomes were reintubation rate and ICU and hospital length of stay, whereas secondary outcome was 1‐year overall survival stratified by MELD score.

**Results:**

Among 158 patients, 47 (29.7%) underwent immediate extubation, with reintubation in one case (2.1%). Immediate extubation was associated with a shorter median ICU stay (4 [IQR 4–5] vs. 5 [IQR 4–5] days, *p* < 0.001) and a nonsignificant reduction in hospital stay (11 [IQR 10–13] vs. 12 [IQR 11–14] days, *p* = 0.084). One‐year OS was higher in the immediate group (95% vs. 80%, *p* = 0.013), with improved survival in MELD 21–30 patients (100% vs. 70%, *p* = 0.047).

**Conclusion:**

Immediate extubation after LDLT appears feasible and safe in carefully selected patients. The observed shorter ICU stay and favorable survival among patients with moderate MELD scores require validation in appropriately matched comparative cohorts.

## 1. Introduction

Prolonged mechanical ventilation following liver transplantation (LT) has been associated with adverse outcomes, including increased risks of pneumonia, sepsis, and multiorgan dysfunction. Historically, postoperative mechanical ventilation with sedation for up to 48 h was considered standard practice, based on the assumption that it conferred hemodynamic stability and facilitated recovery in the immediate postoperative period [1]. However, advancements in perioperative management, such as shorter operative times, the use of short‐acting anesthetic agents, and improved intraoperative techniques leading to reduced blood loss, have paved the way for early extubation strategies in LT [2]. Early extubation has now become a central element of enhanced recovery or “fast‐track” programs in LT.

Despite growing evidence supporting the safety and feasibility of early extubation after LT, routine postoperative mechanical ventilation in the intensive care unit (ICU) continues to be the norm at many transplant centers. Concerns about cardiopulmonary instability, the potential need for surgical reintervention, failed extubation, and suboptimal recovery from surgical stress often drive this conservative approach [3–6].

Living donor liver transplantation (LDLT), in contrast to deceased donor liver transplantation (DDLT), presents unique surgical and physiological challenges. These include the use of smaller grafts and the technical complexity associated with multiple vascular anastomoses [1, 7]. As a result, extubation practices in LDLT have historically been more conservative. Notably, the literature evaluating the safety and efficacy of immediate extubation in the operating room (OR) after LDLT remains limited [1, 6, 8].

Additionally, there is substantial heterogeneity in the definition of “early extubation” across studies, with some defining it as extubation in the OR, whereas others consider extubation within the first hour postoperatively in the ICU [6, 8]. Furthermore, there is a paucity of data evaluating the outcomes of immediate OR extubation specifically in patients with high model for end‐stage liver disease (MELD) scores (> 20), who may have distinct perioperative risk profiles [9].

The objective of this study was to evaluate the feasibility and outcomes of immediate extubation in the OR following LDLT in patients who met predefined intraoperative criteria, including those with MELD scores greater than 20.

## 2. Materials and Methods

This single‐center retrospective cohort study reviewed 227 adult patients who underwent LDLT between February 2023 and May 2025. Following the application of exclusion criteria, 158 patients who met the predefined early extubation criteria were included in the final analysis (Figure 1). These criteria were developed based on consistent trends identified in prior studies, which demonstrated associations between specific intraoperative parameters and the feasibility of early extubation [1, 10, 11]. The standardized criteria for consideration of immediate extubation at the conclusion of surgery included the following:

1. End‐of‐surgery arterial lactate level < 8 mmol/L
2. Requirement of either no inotropic support or a single inotropic agent
3. Intraoperative packed red blood cell (PRBC) transfusion < 7 units

**Figure 1:**
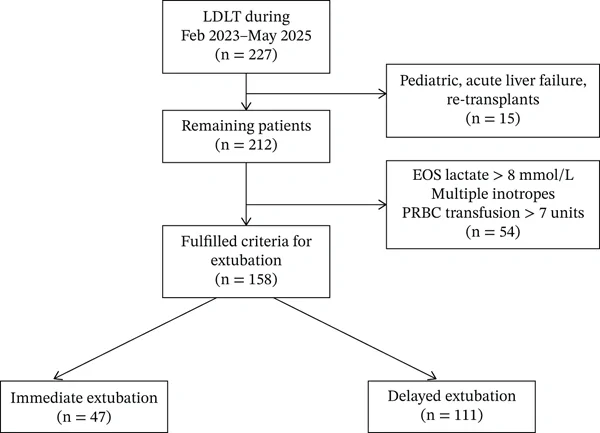
Flow diagram of patient selection and grouping.

Although all patients meeting these criteria were considered eligible for early extubation, the final decision was made at the discretion of the attending anesthesiologist and transplant surgeon. Prior to January 2023, our institutional protocol did not permit OR extubation. Patients were routinely kept intubated postoperatively and remained on mechanical ventilation for a minimum of 12 h, followed by planned extubation on postoperative Day 1 in the ICU. The early extubation protocol was formally implemented in January 2023.

Our center exclusively performs LDLTs, all of which were conducted after approval from the Human Organ Transplant Authority (HOTA). All donor‐recipient pairs were related either biologically or legally, in accordance with national regulations. The details of the donor and recipient evaluation protocols have been previously published [12, 13].

For the purposes of this study, patients were categorized into two groups based on the timing of extubation:

• Immediate extubation group: Extubated in the OR at the end of surgery.
• Delayed extubation group: Extubated in the ICU on postoperative Day 1 or later.

The primary outcomes evaluated included reintubation rates, incidence of pulmonary complications, length of ICU stay, and total hospital stay. The secondary outcome was 1‐year overall survival (OS). Given that the MELD score was not incorporated into the extubation criteria, we also evaluated the feasibility and outcomes of immediate extubation in patients with MELD scores > 20, given their unique perioperative risk profile [9].

Categorical variables were reported as frequencies and percentages, whereas continuous variables were expressed as medians with interquartile ranges (IQR). Statistical analysis of categorical variables was conducted using the chi‐square test, and continuous variables were analyzed using the Mann–Whitney *U* test. Kaplan–Meier survival analysis was used to assess 1‐year OS, and comparisons between groups were made using the log‐rank test. Statistical significance was defined as a two‐sided *p* value < 0.05. The study was approved by the ethics review committee and institutional review board (IRB # 162‐25).

## 3. Results

### 3.1. Patient Characteristics

Pretransplant and intraoperative variables were compared between the immediate and delayed extubation groups, as summarized in Table 1. The majority of baseline characteristics were similar between the groups, with the exception of the MELD score, which was significantly lower in the immediate extubation group (16 [IQR: 13–20]) compared with the delayed group (18 [IQR: 12.7–25]; *p* = 0.031).

**Table 1:** Pretransplant and intraoperative factors in patients with immediate and delayed extubation.

Variables	Immediate extubation (*n* = 47)	Delayed extubation (*n* = 111)	*p*
Pretransplant factors			
Age, median, IQR	50.5 (42.7–56.2)	53 (43–58)	0.498
Male sex, *n* (%)	36 (76.5)	97 (87.3)	0.089
BMI, median (IQR)	25 (23–29.5)	25.4 (22.5–29.7)	0.919
Normal chest X‐ray	37 (78.7)	71 (63.9)	0.068
HCV cirrhosis, *n* (%)	16 (34)	29 (26.1)	0.895
HBV cirrhosis, *n* (%)	16 (34)	34 (30.6)	0.673
MELD, median (IQR)	16 (13–20)	18 (12.7–25)	0.031
MELD > 20, *n* (%)	10 (21.2)	37 (33.4)	0.057
CTP score, median (IQR)	9 (6.7–11.2)	9 (8–11)	0.832
HCC, *n* (%)	3 (6.4)	14 (12.6)	0.399
Intraoperative factors			
Cold ischemia time, median (IQR), minutes	55 (49–70)	58 (49–72.5)	0.540
Warm ischemia time, median (IQR), minutes	61 (53–75)	62 (56–80)	0.381
GRWR, median (IQR)	0.93 (0.85–1.1)	0.98 (0.86–1.14)	0.305
Right lobe graft, *n* (%)	46 (97.9)	109 (98.2)	1.000
Portal vein thrombectomy, *n* (%)	6 (12.7)	22 (19.8)	0.288
Blood loss, median (IQR), mL	1000 (800–1500)	1500 (1000–2500)	< 0.001
PRBC units, median (IQR)	1 (0–1.5)	2 (1–3.25)	< 0.001
FFP units, median (IQR)	0 (0–2)	2 (0–4)	0.002
Surgery duration, median (IQR), minutes	600 (540–660)	660 (600–720)	< 0.001
End of surgery lactate, median (IQR), mmol/L	4 (2.9–5.1)	4.4 (3.4–6.1)	0.166
Max. lactate during surgery, median (IQR), mmol/L	5.2 (4.2–6)	5.6 (4.4–7.4)	0.059
Inotropes, *n* (%)			< 0.001
No inotropes	23 (48.9)	9 (8.1)	
Single inotrope	24 (51.1)	102 (91.9)	
ICU stay, median (IQR), days	4 (4–5)	5 (4–5)	< 0.001
Hospital stay, median (IQR), days	11 (10–13)	12 (11–14)	0.084
Reintubation, *n* (%)	1 (2.1)	7 (6.4)	0.437
Major complications, *n* (%)	11 (23.4)	31 (27.9)	0.556
Pulmonary complications, *n* (%)	2 (4.2)	14 (12.6)	0.091

Intraoperative variables demonstrated statistically significant differences. Patients in the immediate extubation group had lower intraoperative transfusion requirements, shorter operative durations, and reduced need for inotropic support compared with those in the delayed extubation group (all *p* < 0.05).

### 3.2. Primary Outcomes

The median ICU stay was significantly shorter in the immediate extubation group compared with the delayed extubation group (4 [IQR: 4–5] vs. 5 [IQR: 4–5] days, respectively; *p* < 0.001). Although the median hospital stay was shorter in the immediate extubation group (11 [IQR: 10–13] vs. 12 [IQR: 11–14] days), this difference did not reach statistical significance (*p* = 0.084).

The reintubation rate was 2.1% (*n* = 1) in the immediate extubation group and 6.4% (*n* = 7) in the delayed group; however, this difference was not statistically significant (*p* = 0.437). Similarly, pulmonary complications were also less frequent in the immediate extubation group, though not statistically significant (4.2% vs. 12.6%, *p* = 0.091).

### 3.3. Secondary Outcomes

The 1‐year OS for the entire cohort was 84% (Figure 2A). When stratified by extubation timing, the 1‐year OS was significantly higher in the immediate extubation group compared with the delayed group (95% vs. 80%; *p* = 0.013) (Figure 2B). To further assess the feasibility of immediate extubation in patients with higher perioperative risk, subgroup analysis was performed based on MELD scores. As no patients with MELD > 30 underwent immediate extubation, comparisons were limited to two MELD categories: ≤ 20 and 21–30 (Figure 3A). Among patients with MELD ≤ 20, the 1‐year OS was comparable between the immediate and delayed extubation groups (94% vs. 90%, respectively; *p* = 0.376) (Figure 3B). However, in the subgroup with MELD scores between 21 and 30, the 1‐year OS was significantly higher in the immediate extubation group (100% vs. 70%; *p* = 0.047) (Figure 3C).

**Figure 2:**
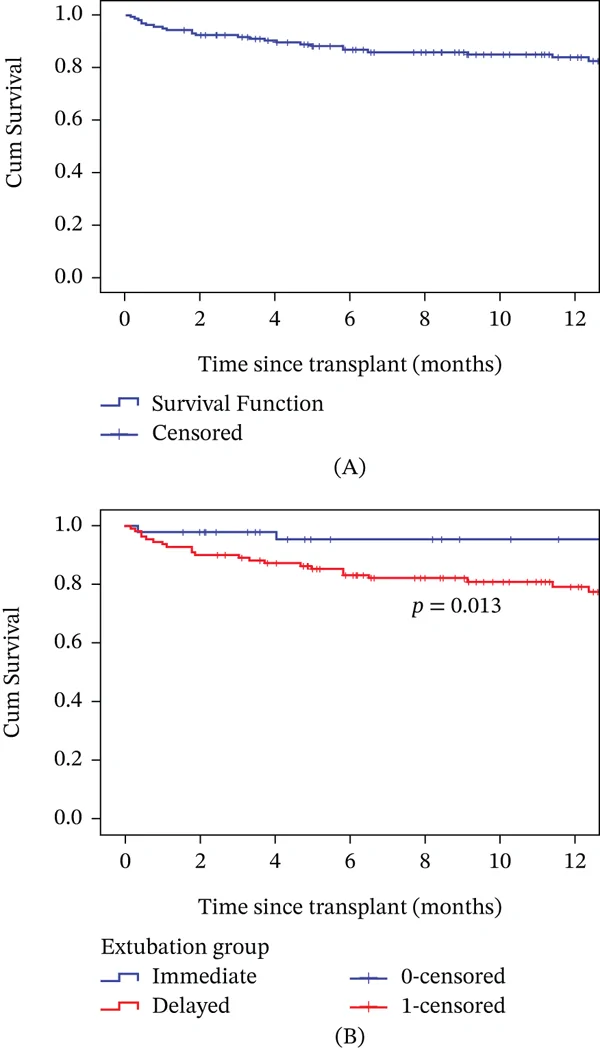
(A) One‐year overall survival in the study cohort (*n* = 158). (B) One‐year overall survival in patients who underwent immediate extubation (*n* = 47) and delayed extubation (*n* = 111).

**Figure 3:**
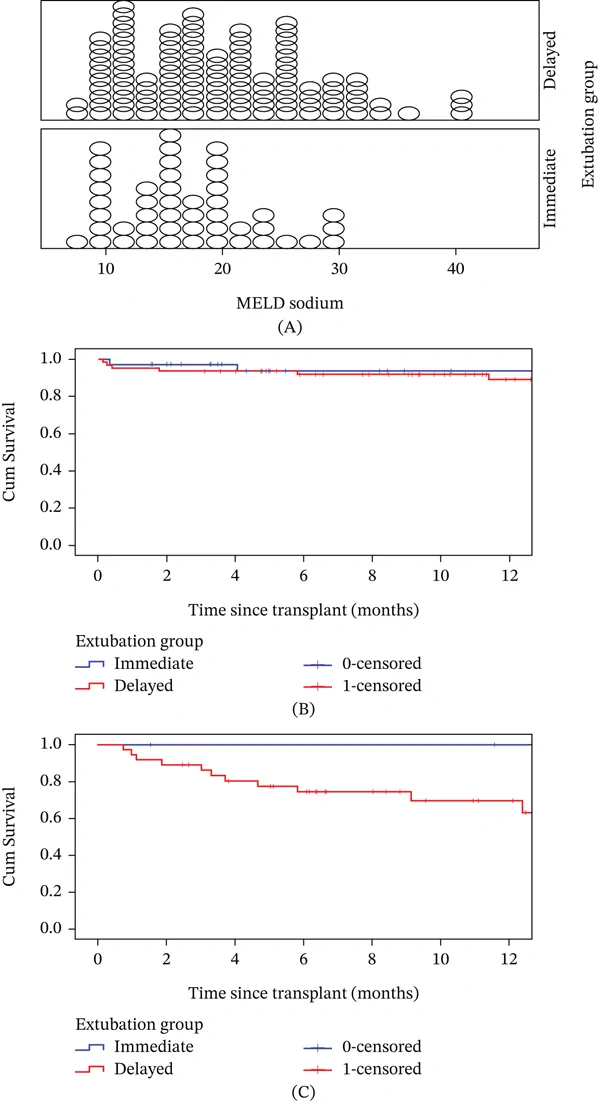
(A) Distribution of MELD‐Na scores in the immediate and delayed extubation groups. (B) One‐year overall survival in patients with MELD‐Na < 20, stratified by extubation group. (C) One‐year overall survival in patients with MELD‐Na 20–30, stratified by extubation group.

## 4. Discussion

Early extubation following LT has gained increasing interest as a strategy to facilitate postoperative recovery and optimize ICU resource utilization [14, 15]. Although most evidence is derived from DDLT, emerging data support its feasibility in LDLT. Our findings suggest that immediate extubation can be safely achieved in carefully selected LDLT recipients, including some patients with moderately elevated MELD scores.

LDLT involves variable surgical complexity, and factors such as smaller graft size, technically demanding vascular reconstruction, use of synthetic conduits, and prolonged operative duration may favor postoperative ventilation [1, 6, 7, 16]. Conversely, the elective nature of LDLT allows greater preoperative optimization and may facilitate early extubation in appropriately selected recipients. In our cohort, 158 of 227 patients (69.6%) met predefined intraoperative criteria for immediate extubation, based on end‐of‐surgery lactate < 8 mmol/L, transfusion of < 7 units of PRBCs, and use of no more than one inotrope. These parameters have been associated with successful early extubation in previous studies [1, 10, 11, 16]. Importantly, MELD score alone may not adequately identify candidates for early extubation; selected patients with MELD > 20 also had favorable short‐term outcomes [5].

Immediate extubation was associated with a significantly shorter ICU stay and a trend toward reduced hospital stay. These findings are consistent with the potential benefits of avoiding prolonged mechanical ventilation, sedation, and their associated complications [17, 18]. Although reintubation rates did not differ significantly between groups, only one patient in the immediate‐extubation group required reintubation for mucus plug–related hypoxia. These findings should be interpreted in the context of careful patient selection.

Immediate extubation was also associated with improved 1‐year OS, particularly among patients with MELD scores of 21–30. Given the retrospective design, this association should not be interpreted as causal. Prior propensity‐matched studies have similarly reported improved postoperative hemodynamics and reduced requirements for organ support following early extubation [19, 20]. These observations may reflect reduced exposure to mechanical ventilation and ICU‐related complications, although residual confounding cannot be excluded [6, 21].

Our findings support early extubation as a feasible, selective component of an LDLT ICU pathway rather than evidence of superiority over standard Day‐1 extubation. Its use remained individualized and was influenced by clinician judgment and practical considerations. The number of immediate extubations did not increase over the study period (19/98 [19.3%] in 2023, 15/99 [15.1%] in 2024, and 13/116 [11.2%] in 2025), suggesting that adoption was not simply related to increasing experience. Extubation was also occasionally deferred despite eligibility, particularly for late‐evening procedures because of overnight staffing considerations.

We avoided comparison with a historical cohort because changes in surgical, anesthetic, and transfusion practices could introduce temporal bias. Eligibility is likely influenced by both modifiable and nonmodifiable factors: Improved intraoperative hemostasis and reduced ischemia times may increase eligibility, whereas patients with severe disease or very high MELD scores may remain unsuitable. Overall, these findings support a carefully selected and individualized approach to immediate extubation after LDLT, guided by preoperative risk, intraoperative stability, and clinical judgment.

This study is limited by its retrospective design and potential selection bias. Despite objective eligibility criteria, institutional factors, including ICU staffing and availability of experienced personnel, may have influenced early extubation decisions, as reported in other centers [1, 5, 15, 22]. Final extubation decisions remained at the discretion of the anesthesiology and surgical teams, introducing potential subjectivity.

## 5. Conclusion

This study supports the feasibility and safety of immediate extubation in carefully selected LDLT recipients, including those with moderately elevated MELD scores. Broader application may be considered with standardized eligibility criteria. The observed shorter ICU stay and favorable 1‐year survival warrant further evaluation in appropriately matched cohorts before attributing these outcomes to early extubation.

**Nomenclature**
DDLT: deceased donor liver transplantation
ICU: intensive care unit
LDLT: living donor liver transplantation
MELD: model for end‐stage liver disease
OS: overall survival

## Author Contributions

Muhammad Nauman‐ul‐Haq did data collection, analysis, manuscript drafting, and statistical interpretation. Muhammad Zakariya Amjad did data analysis, statistical interpretation, and manuscript writing. Haseeb Haider Zia did surgical data acquisition and analysis and final review of the manuscript. Nusrat Yar Khan did surgical data acquisition and analysis and final review of the manuscript. Saad Ahmed Naveed did data interpretation, literature review, and manuscript formatting. Maliha Aziz did data review, analysis, and interpretation. Abid Ilyas did anesthesia data acquisition and protocol standardization. Ayesha Junaid did manuscript writing and revision. Nasir Ayub Khan did senior supervision and critical revision for intellectual content. Abu Bakar Hafeez Bhatti did conceptualization, study design, supervision, manuscript drafting, and critical revision.

## Funding

No funding was received for this manuscript.

## Ethics Statement

The study was approved by the ethics review committee and institutional review board (IRB # 162‐25).

## Conflicts of Interest

The authors declare no conflicts of interest.

## Data Availability

The data that support the findings of this study are available on request from the corresponding author. The data are not publicly available due to privacy or ethical restrictions.
